# An Exposure of Quackery: The Select Committee's Report on Patent Medicines

**Published:** 1914-09-12

**Authors:** 


### AN EXPOSURE OF QUACKERY.

### The Select Committee's Report on Patent Medicines.

The recently issued Report of the Select Committee on Patent Medicines makes its appearance at a time when the newspaper press has room for only one topic, and the result is that the Report has been allowed to pass almost unnoticed. All the information that has been furnished to the general public concerning the matter has been contained in the briefest of paragraphs, and consequently few people are aware that a powerful and fearless condemnation of that form of quackery which manifests itself in secret remedies has been presented to the House of Commons by a strong Committee representative of all parties. There are possibly some who hold the view that at no time would the lay press have found room for an adequate summary of the Report; but this view is hardly a just one, for notwithstanding the £2,000,000 a year that is said to be spent on the advertising of proprietary articles, it cannot be believed that the press would willingly ignore a document that has so intimate a bearing upon the public health, and which demonstrates conclusively that the sale of some of the most largely advertised and best-known articles depends to a large extent upon the exaggerated or fraudulent claims which their proprietors make for them.

Sooner or later the recommendations of the Committee will be embodied in a Parliamentary measure, and although strong opposition on the part of vested interests is anticipated, nothing can prevent the enactment of legislation designed to put a stop to the gross abuses which the Report has exposed. At present the law is powerless to prevent "any person from procuring any drug, or making any mixture, whether potent or without any therapeutical activity whatever (so long as it does not contain a scheduled poison), advertising it in any decent terms as a cure for any disease or ailment, recommending it by bogus testimonials and the invented opinions and facsimile signatures of fictitious physicians, and selling it under any name he chooses, on the payment of a small stamp duty, for any price he can persuade a credulous public to pay." The traffic in secret remedies is thus practically uncontrolled in this country, and this, in the words of the Report, is "an intolerable state of things."

Briefly, the Report divides proprietary medicines into two main classes—namely, non-secret medicines and secret medicines. Against the first class few objections are urged, but the case against the second class is unanswerable. This second category is subdivided into four groups—to wit, (1) simple household remedies; (2) dangerous drugs and drugs for improper purposes; (3) fraudulent remedies; (4) remedies making grossly exaggerated claims. It is the last three groups that cause the bulk of the mischief, and the remedies which they comprise constitute a grave and widespread public evil. They cause injury by leading sick persons to delay in securing medical treatment; some of them contain in disguise large proportions of alcohol; others profess to cure diseases that are incurable by medication; and others are essentially or deliberately fraudulent. None of them springs from therapeutical or medical knowledge, and, as the Report says, "they are put upon the market by ignorant persons, and in many cases by cunning swindlers who exploit for their own profit the apparently invincible credulity of the public." To protect the public from these swindlers the Committee proposes legislative action of a kind which would not inflict injustice upon any patent or proprietary medicine, although it would ruin many of the proprietors who, if justice had prevailed, would have been ruined long ago. The proposals of the Committee are comprehensive. They do not recommend the exhibition of formulæ, for several reasons; any benefit resulting from a label stating the exact composition of a remedy must depend for its efficacy upon the intelligence and education of the intending purchaser, and the disclosure that a remedy contains "acetyl-salicylic acid," or "hexamethylene-tetramine," or "phenolphthalein," or "acetanilide" would be meaningless to most people. Then, again, the simplest substance might acquire distinction from being described in technical language—soap, for instance, a large ingredient of the most popular aperient pills, posing as "sodium oleate and stearate." For these, among other reasons, the Committee do not think the exhibition of formulæ a practical or effective measure.

They recommend, however, that the formulæ of all secret remedies should be required to be communicated to a competent officer appointed under the authority of a Minister of State—a Ministry of Public Health is suggested—but in the meanwhile the duty could be undertaken by the Local Government Board. It is also proposed that a special Commission be constituted, with power to permit or to prohibit in the public interest the sale and advertisement of any secret medicine. Another recommendation is that the advertisement and sale of medicines purporting to cure the following diseases be prohibited: Cancer, consumption, deafness, lupus, diabetes, paralysis, fits, epilepsy, locomotor ataxy, Bright's disease, and rupture (without operation or appliance); further, it is proposed to prohibit all advertisements of diseases arising from sexual intercourse or referring to sexual weakness, and all advertisements likely to suggest that any medicine is an abortefacient. With regard to medicated wines, the Committee recommend that the makers of every such wine be required to state upon the label the proportion of alcohol contained in it. Certainly no measures of smaller scope than those suggested will afford the public efficient and urgently needed protection against injury and fraud, and it is to be hoped that the enactment of legislation of the kind proposed will not be delayed unnecessarily.

